# NOX4: a potential therapeutic target for pancreatic cancer and its mechanism

**DOI:** 10.1186/s12967-021-03182-w

**Published:** 2021-12-20

**Authors:** Yawei Bi, Xiao Lei, Ningli Chai, Enqiang Linghu

**Affiliations:** 1 Department of Gastroenterology and Hepatology, The First Medical Center of PLA General Hospital, Beijing, 100853 China; 2 Senior Department of Oncology, The Fifth Medical Center of PLA General Hospital, Beijing, 100859 China

**Keywords:** NADPH Oxidase 4, Pancreatic cancer, Reactive oxygen species

## Abstract

Nicotinamide adenine dinucleotide phosphate (NADPH) oxidase 4 (NOX4) is one of the seven isoforms of NOX family, which is upregulated in pancreatic cancer cell, mouse model of pancreatic cancer and human pancreatic cancer tissue. NOX4 is a constitutively active enzyme that primarily produces hydrogen peroxide, which exhibits completely different properties from other subtypes of NOX family. More importantly, recent studies illuminate that NOX4 promotes pancreatic cancer occurrence and development in different ways. This review summarizes the potential roles and its mechanism of NOX4 in pancreatic cancer and explores NOX4 as the potential therapeutic target in pancreatic cancer.

## Introduction

Pancreatic cancer (PC) is a highly malignant digestive system tumor with a poor prognosis. Despite advances in medicine has improved the survival rate of many types of aggressive cancer, PC remains one of the deadliest malignancies with an incidence/mortality ratio of as high as 94% and a 5-year survival rate of about 9% [1]. It is predicted that PC will rise to the second-leading cause of cancer-related deaths in the U.S. in 2030 [2]. Therefore, it is urgent for us to explore the pathogenesis of PC and search its potential therapeutic targets. PC has a complex landscape of genetic alterations with prevalent chromothripsis and mutations in KRAS, TP53, SMAD4, and CDKN2A [3–5]. However, heterogeneous and complex compositions of genetic alterations may, in concert, drive common phenotypes that expose specific vulnerabilities. One such phenotype that has emerged as a potential vulnerability in cancer is aberrant redox homeostasis [6].

Reactive oxygen species (ROS) exerts oxidative stress in the cells which deregulate the redox homeostasis and promote tumor formation by initiating an aberrant induction of signaling networks that cause tumorigenesis [7]. ROS is a by-product of cell metabolism, including superoxide anions, hydroxyl radicals and hydrogen peroxide. Nicotinamide Adenine Dinucleotide Phosphate (NADPH) oxidases are a major intracellular source of ROS, and evidence suggests that ROS production by NADPH species can strongly influence both tumor growth and survival [8, 9]. Over the decades, six human homologs of the catalytic subunits of the phagocyte NOX were found: NOX1, NOX3, NOX4, NOX5, DUOX1 and DUOX2.Together with the NOX2/gp91phox component present in the phagocyte NADPH oxidase assembly itself, the homologs are now referred to as the NOX family of NADPH oxidases. The isoforms of NOX are distributed in different tissues, cells and subcellular structures, produce corresponding products, and perform specific functions under physiological and pathological conditions [10].

NOX4 is identified as a nonphagocytic novel NOX in kidney in 2001. Then researchers found that NOX4 is expressed in not only kidney tissues, but also lung, ovarian, pancreas and other organs [11–13]. Unlike other isoforms, NOX4 is a constitutively active enzyme. It produces H_2_O_2_ as the sole or vast majority of detectable ROS product even in vitro in the absence of superoxide dismutase [14]. Compared with other members of the NOX family, NOX4 widely expressed in many different tissues and has a wider range of biological functions. As the main endogenous ROS source, NOX4 is involved in regulating multiple functions of cells including cell proliferation, migration and death [15]. NOX4 generates lower level of ROS which serve as second messengers to induce a panel of intracellular signaling pathways such as HIF2-α, p38 MAPK, TGF-β1/Smad2/3, Akt, Caspase3 to regulate functions of cells [15]. However, when some stimulation signals are received, NOX4 could generate high level of ROS and cause the activation of multiple downstream signal pathways which may promote disease development. Preliminary immune-histological and gene expression surveys of human primary tumor samples have revealed elevated NOX4 protein or transcript levels relative to adjacent normal healthy tissues in several tumor types [11, 16, 17]. The data from gene and protein profiling, cell lines, mouse model and human PC tissues suggest that NOX4 expression is significantly increased in PC which reminds us that NOX4 may play a vital role in the development of PC [18, 19]. Previous studies have shown that NOX4 participates in PC progression via different ways [20–22]. However, the specific mechanism of NOX4 in the occurrence and development of PC remains unclear. In this review, we summarize the currently available evidence regarding the role and the mechanism of NOX4 in PC and its possibility as a potential target for the treatment of PC.

## NOX4 is involved in the pancreatic tumorigenesis

The human NOX4 gene comprises 18 exons and is located on chromosome 11q14.2-q21 [23]. NOX4 is quite different from other NOX isoforms as it primarily produces H_2_O_2_ due to a unique third extracytosolic loop (E-loop) [14]. Of the multiple types of ROS, H_2_O_2_ is an adept signaling molecule. H_2_O_2_ is a relatively stable oxidant that is able to cross membranes and react with protein thiol moieties to produce post-translational modifications, altering protein function [[12]. Recent studies showed NOX4 was closely associated with the occurrence and development of different cancers [24–26]. Moreover, NOX4 was upregulated in PC [18, 21]. Therefore, we consider that NOX4 plays a vital role in PC progression. The potential mechanisms of NOX4 in PC are as follows.

### NOX4 regulates PC cells from death

One reason why PC is so aggressive and unresponsive to treatments is its resistance to apoptosis. Previous research showed that the antiapoptotic effects of growth factors in PC cells are mediated via ROS produced by NOX4 [27]. Moreover, Mochizuki pointed out that ROS generated by NOX4 transmit cell survival signals through the AKT-ASK1 pathway and Jong showed that NOX4 generated ROS promote PC cell survival via inhibiting JAK2 dephosphorylation by tyrosine phosphatasesin [28, 29].

Cell senescence is the process by which cells stop dividing and lose their ability to proliferate. Therefore, cellular senescence plays a crucial role in suppressing cancer [30]. ROS have been proposed to be signaling molecules that mediate proliferative cues. However, ROS may also cause DNA damage and proliferative arrest. How these apparently opposite roles could be reconciled, especially in the context of oncogene-induced cellular senescence, which is associated both with aberrant mitogenic signaling and DNA damage response (DDR)-mediated arrest, is unclear. Ogrunc et al. showed that NOX4 promotes transformation of oncogene-expressing PC cells by generating mitogenic ROS, and transformed cell cause inactive DDR and oncogene-induced cellular senescence bypass [19]. Therefore, NOX4-dependent ROS are indeed mitogenic signaling molecules that fuel oncogene-driven aberrant cell proliferation in PC.

### NOX4 promotes fibrosis in PC

In the occurrence and progression of PC, the most significant feature is the formation of pancreatic fibrosis. In this process, pancreatic fibrosis creates a microenvironment of hypo perfusion, hypoxia, and immune shielding in pancreatic tissue, which brings treatment more difficult [31]. NOX4 has been proven to play an important role in the fibrosis process of different organs. Zhao et al. found upregulation of NOX4 in the myocardium causes cardiac remodeling through activating Akt-mTOR and NF-κB signaling pathways [32]. In fibrotic lung disease, NOX4 expression is increased and plays a deleterious role by reducing fibroblast apoptosis, leading to fibroblast accumulation and fibrosis progression [33]. In kidney, alcohol promotes renal fibrosis by activating NOX4-mediated DNA methylation of Smad7 [34].

The key to pancreatic fibrosis is the activation of pancreatic stellate cells (PSCs). Although previous studies found that NOX enzymes play a crucial role in the activation of PSCs and extracellular matrix (ECM) formation [35–37], the specific role of NOX4 in the process of pancreatic fibrosis has not been extensively studied. While it is worth noting that the process of pancreatic fibrosis is similar to liver fibrosis. Like pancreatic fibrosis, the key of hepatic fibrosis is closely related to the activation of hepatic stellate cells (HSCs). Normally, PSCs or HSCs are quiescent and regulate ECM production. However, PSCs and HSCs are activated by many stimulating factors during tumorigenesis [38, 39]. Then, active PSCs or HSCs can create a suitable microenvironment and facilitate cancer progression by altering four processes in hepatic cancer or PC models: excessive fibrosis, promoting tumor metastasis, inducting resistance of chemotherapy and radiotherapy and immune modulation [40, 41].

Plenty of researches already proved that NOX4/ROS signaling pathway promotes the proliferation and activation of HSCs [42–46]. Moreover, experiments using siRNA against NOX4 attenuated HSCs activation, and more importantly, knocking down NOX4 in activated myofibroblasts could reverse the fibrotic phenotypes [47, 48]. In summary, NOX4 is involved in fibrosis of different organs, especially in liver. And considering the similarity between the process of pancreatic fibrosis and liver fibrosis, we believe that NOX4 also plays a key role in pancreatic fibrosis. However, the role of NOX4 in pancreatic fibrosis deserves further study.

### NOX4 contributes to EMT in PC

The epithelial-mesenchymal transition (EMT) process is a major contributor to the development of resistance in multiple cancer types including PC [49]. Classical EMT involves a phenotypic change in cells, in which cells loss their epithelial phenotype, such as tight cell-to-cell adhesion and apical-basal polarity, and acquire a highly invasive, mesenchymal phenotype [50]. Accumulating evidence suggest that EMT plays an important role in the pathogenesis, invasion, metastasis, and drug resistance in PC [51–53]. Notably, many key EMT regulators were recently found to be redox-sensitive, enabling the elucidation of the molecular basis of EMT from a redox perspective. ROS, an important cellular secondary messenger containing free radical species, can alter the biological functions of redox-sensitive proteins involved in ECM remodeling and cell mobility, thereby regulating EMT [54, 55]. One of the primary sources of ROS production is via NOX enzymes and previous studies proved NOX4 is involved in the EMT process in different organs such as lung, kidney, liver, cervix and breast [24, 56–59].

The role of NOX4 in the EMT process of PC has also been studied. Ma et al. showed that NOX4 mRNA correlation with EMT gene expression such as collagen (COL1A2, COL3A1, COL5A2), metalloproteases (MMP2, MMP9) and fibronectin (FN1) [21]. Risako et al. treated the PC cells with the NOX4 inhibitor diphenylene iodonium and NOX4 siRNAs, the results showed downregulation of NOX4 blocked TGF-β-induced EMT phenotype including morphological changes, augmented migration, and altered expression of E-cadherin and Snail in PC cells, which showed that NOX4 transmit TGF-β-triggered EMT signals in PC [11]. David et al. suggest that TGF-β1-induced EMT in PC cells is mediated through RAC1/NOX4/ROS/p38 MAPK cascade [60]. Recent research showed that NOX4 caused inactivation of lysine demethylase 5A, increased the methylation modification of histone H3 and regulated the transcription of EMT associated gene SNAIL1. And NOX4 deficiency repressed hypoxia-induced EMT in PC cells [61]. To sum up, NOX4 and NOX4-mediated ROS generation play vital roles in regulating EMT process in PC.

### NOX4 plays a vital role in metabolic regulation

Metabolism reprogramming is the hallmark of tumor cells, and it has a causal relationship with the occurrence and development of tumors. Regulated metabolic changes in tumor cells include aerobic glycolysis (the Warburg effect), increased glucose uptake, abnormally active glutamine metabolism, and the use of non-primary energy-supply substances for energy supply [62]. These metabolic changes satisfy the rapid growth of tumor cells. The strong energy and material requirements during proliferation help cells adapt to the hypoxic tumor microenvironment, and then provide energy and material support for tumor proliferation, invasion, migration and other biological activities [63].

NOX4 has been reported as a glycolytic regulator in different tissues. Tang et al. used papillomatosis thyroid cancer cells to study the cell growth by knocking down the expression of NOX4 and knocking out its functional partner p22phox/CYBA, the results suggested that NOX4 participated in regulating glycolysis through mROS-HIF1α pathway, thereby mediating proliferation in thyroid carcinomas [26]. In terms of human neuroblastoma cells, it is showed that knockdown of NOX4 expression by siRNA inhibited glycolysis induced by hypoxia through decreasing the expression of glycolysis-related proteins (HIF-1α, LDHA, and PDK1), decreasing glucose uptake, lactate production, and ROS production, while increasing mitochondria membrane potential [64]. Zeng et al. revealed NOX4 promotes glycolysis, contributing to non-small cell lung cancer growth, and supports glutaminolysis for oxidative resistance [65]. David et al. proved that disturbed flow could increases NOX4 and ROS to stabilize endothelial HIF-1α which stimulates glycolysis in endothelial cells and then results in vascular inflammation and ultimately atherosclerosis [66]. In respect to the metabolic change in PC, Ju et al. showed that elevated NOX4 activity accelerates oxidation of NADH and supports increased glycolysis by generating NAD^+^, a substrate for GAPDH-mediated glycolytic reaction, promoting PDAC cell growth [18].

With more and more evidence showing that NOX4 mediates the metabolic reprogramming in different organs, the role of NOX4 in the regulation of PC cell metabolism and its mechanism deserve further exploration. Firstly, more in vivo experiments are needed to confirm that NOX4 could participate in the regulation of PC metabolic changes. Then, the specific mechanisms by which NOX4 regulates PC metabolism changes should be studied, such as whether they play roles in interfering with glycolysis-related proteins or activating certain downstream signaling pathways. Finding out the specific mechanisms how NOX4 regulates PC metabolic changes would help us make better strategies for PC treatment.

### NOX4 mediates angiogenesis in PC

Angiogenesis is necessary for the invasive growth and metastasis of tumors and is an important target in the control of cancer progression [67]. Despite of conflicting views about the formation and recruitment of new blood vessels in human PDAC [68, 69], decades of studies demonstrate that PDAC, like other cancers, need new and destabilized blood vessels (tumor angiogenesis) as a prerequisite event for the growth and progression as well as dissemination of tumor cells for metastasis [70, 71].

Angiogenesis is a tightly regulated multistage process, including vessel sprouting, lumen formation and maturation [72]. Upon pro-angiogenic stimulation, endothelial cells (ECs) firstly sprout from the pre-existing vascellum after the degradation of ECM. Afterwards, these ECs undergo proliferation, migration and differentiation and recruit smooth muscle cells (SMCs) or pericytes to cover the newly-formed vessels to promote their maturation. The essential role of NOX4 in angiogenesis has been the subject of research for years. NOX4 is the major isoform of NADPH oxidases expressed in vascular cells and predominantly produce ROS, which play an important role in angiogenesis. In femoral artery ligation mice model, NOX4-/- mice exhibit attenuated angiogenesis, while endothelial-specific NOX4 transgenic mice exhibit enhanced angiogenesis and blood flow recovery under ischemia in an eNOS-dependent manner [73, 74]. Not only is involved in a variety of physiological processes, NOX4 also mediates angiogenesis in pathological conditions which can cause cancer.

Compelling evidence demonstrates that NOX4 and its generated ROS have a close relation to tumor angiogenesis in different cancers. In a carcinogen 3-methylcholanthrene (MCA)-induced fibrosarcoma mice model, NOX4 was proved to regulate the tumour-vessel density through stabilization of HIF-1α and induction of VEGF expression, while a significant 38% reduction in tumour vascularization in fibrosarcomas of Nox4-/- mice [75]. In von Hippel Lindau (VHL)-deficient renal cell carcinoma, NOX4 also promotes renal tumorigenesis in a similar signal pathway via nuclear accumulation of HIF-2α [76]. Li et al. showed that stable NOX4 knockdown reduced ROS production significantly and suppressed glioblastoma cells proliferation and invasion and tumor associated angiogenesis [77].

HIF-1 is a key transcription factor of angiogenesis in solid tumors including PC and NOX4 can regulate angiogenesis through HIF-1 in different cancers [78, 79]. Thus, we assume that NOX4 may mediate angiogenesis in a similar way to promote the development of PC which need more studies to prove that.

## Perspective: Targeting NOX4 for the therapy of pancreatic cancer

As NOX4 participates in the progress of PC via different ways, it has been emerging as a promising therapeutic target for PC treatment. We believe more studies are worth to do in some aspects. As discussed above, oncogenes mutation drives the development of PC. However, the carcinogenic mechanism of these mutant oncogenes remains exclusive. Recent studies showed that some oncogenes promote PC progress through interacting with NOX4. *TP53* mutations could “switch” NOX4 from being protective and an indicator of good prognosis to deleterious by promoting programs favoring cancer progression including EMT, cell migration, cell adhesion, and angiogenesis [21]. Ju et al. found that NOX4 is the key point of interaction between *KRAS* activation and *P16* inactivation to promote the occurrence of pancreatic cancer [18]. According to the full exome group sequencing of PC, *KRAS*, *TP53, CDKN2A* and *SMAD4* are most common oncogenes. Therefore, more studies are needed to explore the relationship between NOX4 and other oncogenes, such as *CDKN2A* and *SMAD4*. If we could prove that a variety of genetic mutations play carcinogenic role in PC are associated with NOX4, then NOX4 would become the greatest target of PC as it could be applied to patients carrying different genetic mutations.

**Fig. 1:**
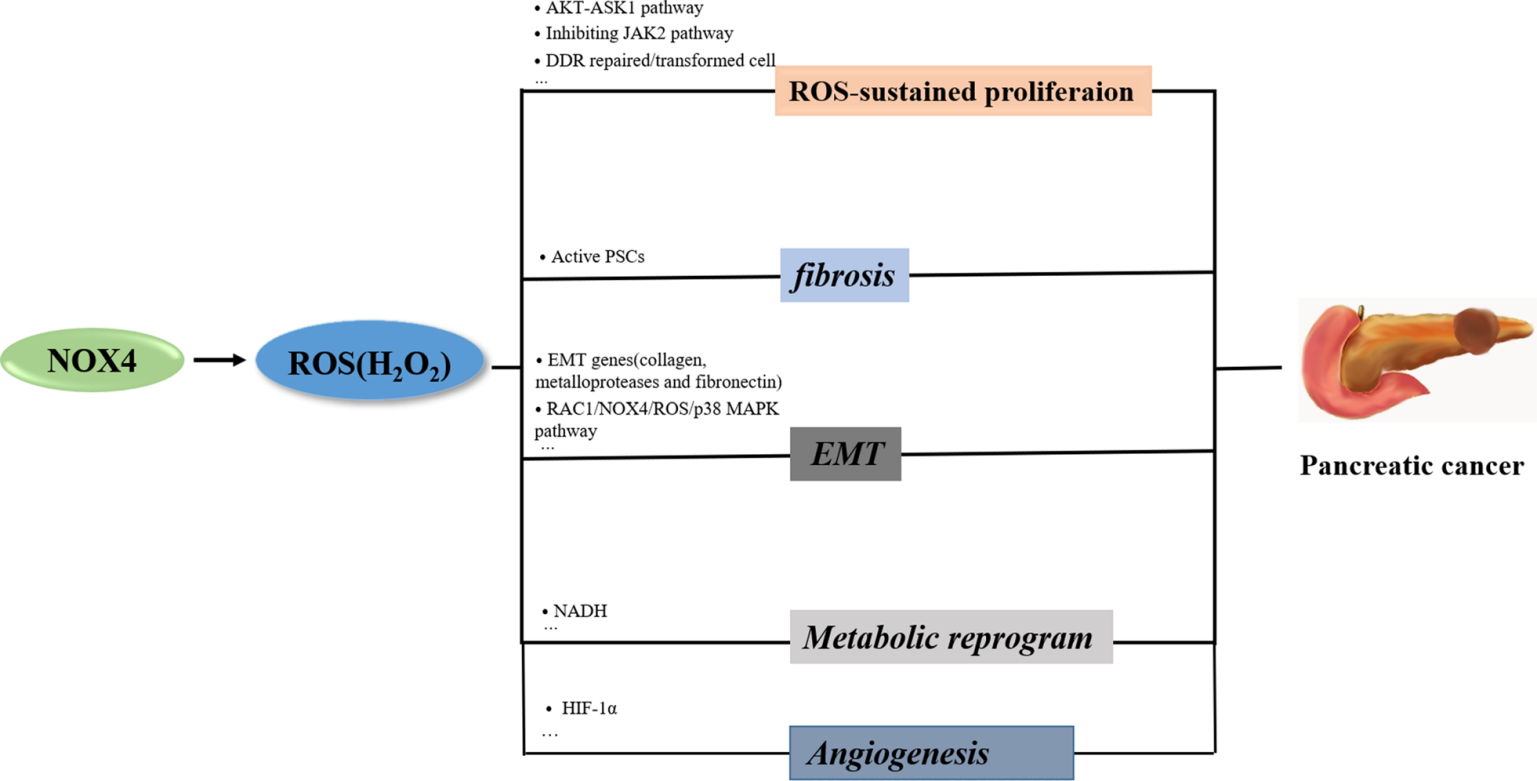
The scheme of the potential roles of NOX4 in pancreatic cancers

As presented in this review, NOX4 can participate in the development of PC via different mechanism, which prove that it could be a promising target for the PC treatment (Fig. 1). Although some studies have shown that pharmacological/genetic inhibition of NOX4 can inhibit tumor development at the cellular level or in animal models, the effectiveness in human still needs further clinic trial [20, 22]. This is the second point we could explore.

Recent breakthroughs in cancer treatment consisting of new combinations of existing medications. Drug resistance is the main reason why chemotherapeutics cannot achieve the desired therapeutic effect. Interestingly, NOX4 may related to chemotherapy resistance [80, 81]. Karthigayan et al. found NOX4 functions as a mitochondrial energetic sensor coupling cancer metabolic reprogramming to drug resistance [82]. In ovarian cancer cells, NOX4 knockdown increased sensitivity of targeted therapy and radiotherapy through decreased expression of HER3 and NF-κB p65 [12]. Ju et al. tested the impact of NOX4 inhibitors combined with gemcitabine on Panc1 cells-a human PDAC cell line carrying a mutant K-Ras allele.53, and observed that the combination therapy displayed a strong synergistic impact on reduced cell viability and enhanced apoptosis and it increased the half-maximal inhibitory concentration of gemcitabine by four- to sixfold [19]. These results indicated us that chemotherapy with NOX4 inhibitor may achieve better therapeutic effects in PC. This is the third and most important idea which we could test.

Therefore, research needs to further explore the role of NOX4 in PC progression and chemotherapy resistance, so that clinic can make individual treatment for PC patients to enhance drug efficacy, extending patient survival, and improve quality of life.

## Conclusion

Despite rapid advances in modern medical technology and significant improvements in survival rates of many cancers, PC is still a highly lethal gastrointestinal cancer with a low 5-year survival rate and difficulty in early detection. Exploring the pathogenesis of PC and seeking its therapeutic targets has become an urgent issue.

NOX4 is one of the NOX family. Unlike most other subunits generating O_2_^−^, NOX4 catalyzes the reduction of molecular oxygen to H_2_O_2_. This feature gives NOX4 some specific roles in different cellular functions, such as proliferation, differentiation, migration, apoptosis, senescence and matrix secretion [83–88]. NOX4 has been proven to participate in the development of PC by promoting cell proliferation, activation of PSCs, EMT progression, regulating cell metabolism changes and mediating angiogenesis. However, its specific mechanism of these effects is still exclusive and awaits further study. Since NOX4 is increased in PC and promotes PC development via variety ways, it may be a robust potential therapeutic target for PC. More researches also need focus on the therapeutic effect of NOX4 in PC.

## Data Availability

The datasets are available under reasonable request.
